# Evaluation of Composite Resin Bonding to Coronal Dentin Contaminated by Endodontic Sealers: Evaluation of Composite Resin Bonding by Endodontic Sealers

**DOI:** 10.31661/gmj.v13iSP1.3680

**Published:** 2024-12-30

**Authors:** Parya Atapour, Mehdi Daneshpooy, Fatemeh Pournaghiazar, Reza Safaralizadeh

**Affiliations:** ^1^ Department of Oral and Maxillofacial Medicine, Faculty of Dentistry, Tabriz University of Medical Sciences, Tabriz, Iran; ^2^ Department of Operative Dentistry, Faculty of Dentistry, Tabriz University of Medical Sciences, Tabriz, Iran

**Keywords:** Shear Strength, Mineral Trioxide Aggregate, Root Canal Filling Materials, Epoxy Resin AH-26, Zinc Oxide-Eugenol

## Abstract

**Background:**

Endodontically treated teeth lose their structure primarily as a result of trauma, decay, and during root canal therapy. Root canal sealers containing eugenol reduce the bond strength of resin cements, therefore present study investigates the shear bond strength of composite to dentin contaminated by endodontic sealers using three types of sealers.

**Materials and Methods:**

In this study, 60 human premolar teeth crowns were cross-sectioned to expose the coronal dentin. The samples were divided into 4 groups of 15. In the 3 groups, the dentin surface was contaminated by Endofill, AH26, and MTA Fill apex sealers respectively and group 4 was considered as a control group. The specimens’ shear bond strength was measured by a universal testing machine with a loading speed of 1mm/min. The mean shear bond strength was analyzed using Kruskal-Wallis and U Mann-Whitney by SPSS 16. P0.05 was considered significant.

**Results:**

The mean shear bond strength of the studied groups was significantly different (P=0.03). The highest shear bond strength was seen in the control group and the lowest one was related to the Endofill group. A significant difference was seen between the shear bond strength of the two groups (Endofill, AH 26) (P=0.02) and (Endofill, control) (P=0.01).

**Conclusion:**

The contamination of dentine with endodontic sealers significantly reduces the shear bond strength of composites to dentin. The shear bond strength was lowest in eugenol-based sealer.

## Introduction

Endodontically treated teeth lose their structures mainly due to trauma, caries, and endodontic treatments [1][2]. Restoration of endodontic teeth is critical for achieving clinical success [3].

Root canal sealers are essential for sealing the space between the dentin wall and the main cone. Sealers also fill bubbles and root canal irregularities, accessory and lateral canals, and the space between the gutta-percha cones used in lateral compression [4].

Zinc oxide-eugenol-based sealers are widely used in dentistry due to characteristics such as fast setting time [5]. Numerous studies have indicated that eugenol-containing sealers can reduce the bond strength of resin cement [6][7][8]. Following the endodontic treatment, teeth often need extensive restorations and buildup using composite resins and a dentin adhesive [9].

Decreased shear bond strength of composite to dentin can be observed in full crowns using, Zinc Oxide-Eugenol (ZOE) temporary cement [10]. Resin sealers are the new generation of sealers known as Monoblock that can be attached to the dentin and core material [11].

Mosharraf et al. examined the effect of endodontic sealers on the bond strength of fiber-post to the root dentin and found that tensile bond strength was significantly higher in the AH26 sealer group (resin-based) than in the group with Endofill (a eugenol-containing sealer) [12]. Aleisa et al. also studied the effect of three sealer types on the bond strength of fiber-post with resin cement to root dentin and observed that bond strength in the group with Endofill and Tubli-Seal sealers (eugenol-containing sealers) was significantly lower than the AH26 sealer group [6].

Recently, MTA-based sealers have been introduced to achieve suitable biological properties and proper seals [13]. Forough Reyhani et al. reported that resin-based sealers had the highest push-out bond strength compared with ZOE- and MTA-based sealers [14]. Some studies investigated the bond strength of resin cement to root dentin, but no study has focused on the effect of different sealers on the shear bond strength of resin composite to coronal dentine. Therefore, the present study aimed to investigate the shear bond strength of resin composite to crown dentin contaminated with three endodontic sealers.

## Materials and Methods

**Figure-1:**
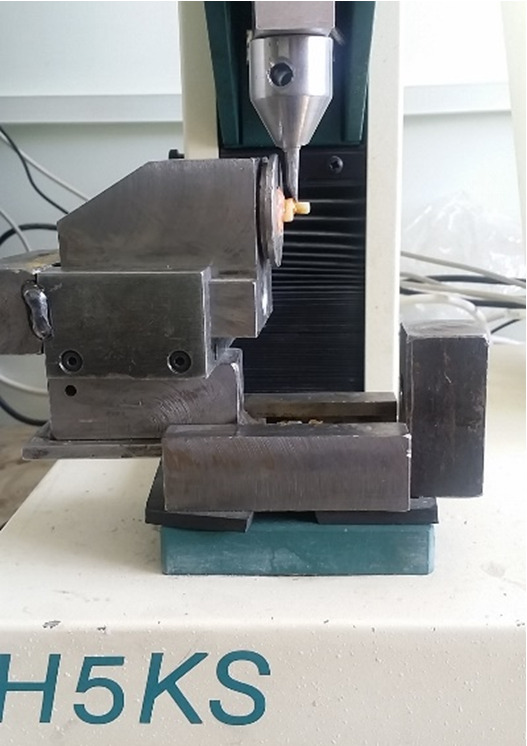
Figure 1. Universal testing machine conducting shear bond strength test

In this experimental study, 60 healthy human premolars were used. Based on the study of Mosharraf et al., [12] a difference of 1.44 was estimated between the mean bond strengths of the two groups. A total of 13 samples were obtained according to α=0.55, a power of 80%, and a between-group difference of 0.75. To increase the validity of the study and due to possible loss of samples, each group consisted of 15 samples. Healthy extracted human premolars without abrasions or cracks were included in the study and teeth with previous restoration, endodontic treatment, internal discoloration, and cracks were excluded.

In this in vitro study, 60 cylindrical acrylic specimens, using pink acrylic (Triplex, Ivoclar Vivadent, Liechtenstein) were prepared and the teeth were then placed inside the acrylic. Samples were cut transversely by a trimmer (Kavo Electrotechnisches Werk, type 5404, West Germany) to expose the deep coronal dentin.

The occlusal surface of the samples was polished with 320-grit silicon carbide papers (Soflex, 3M ESPE, ultra-thin, USA) and then divided into four groups of 15. The samples were randomly assigned to one of the groups, using Randlist software,

In the first group, the dentin surface was contaminated with an MTA Fillapex sealer (Angelus, Londrina, PR, Brazil) as a uniform layer by a micro brush and then the surface was covered with tinfoil. Then the samples were placed vertically in a lid plastic container. The container was poured with 1 cm of water, its lid was closed tightly, and the container was kept at 37℃ for 6 days.

The dentin surface was then mechanically cleaned with a carving instrument. The samples were etched with 35% phosphoric acid gel (Scotchbond Etchant, 3M, Dental products St, Paul, MN, USA) for 15 s, washed with water for 30 s, and then air-dried without water and oil contamination for 5 s. In the next step, a one-bottle adhesive Adper single bond (3M ESPE, Dental products ST, Paul, MN, USA) was applied on the prepared surface of the samples using a clean micro brush (Microbrush Co., Greyton, W1, USA). According to the manufacturer, this material was applied in two layers and, after adding the second layer, the solvent was evaporated through gentle air-drying for 2-5 s. The adhesive layer was then light cured for 20 s by an Astralis device (Ivoclar Vivadent, FL Schaan) adjusted to a low-power program with a constant intensity of 400 m/cm2. To make the cross-section of the composites uniform in all samples, transparent molds with a diameter and height of 3 mm were used, which were placed on the prepared samples, and the composite (Filtek Z250 (3M_ESPE Dental Products, ST. Paul, MN, USA) with A2 color were packed in two layers by condenser inside the clear molds, the layers were 1.5 mm thick and placed horizontally and the thickness of each layer was measured with a probe and then each layer was cured for 20 s from the occlusal side before adding the next layer. Finally, after curing the second layer, the entire composite mass was cured from the sides for 40 seconds.

The samples were kept at 37℃ for 24 h and then exposed to 1000 thermal cycles at 5-55℃. The shear bond strength of the samples was measured by a universal testing machine (Hounsfield 5k, UK, England) using a chisel-shaped blade tangential to the composite and the tooth interface at a loading speed of 1 mm/min Figure-1). The force was applied until the moment of fracture. Eventually, each tooth diagram was recorded by a computer.

The procedure followed in the second and third groups was similar to the first group, except that the AH26 sealer (Dentsply Detray GmbH, Konstanz Germany) and the Endofill sealer (PD, Switzerland Swiss) were used respectively. The fourth group was the control with no sealer used.

## Statistical analysis

The mean shear bond strength and standard deviation were calculated for each of the experimental groups. Next, the obtained data were analyzed using Kruskal-Wallis and Mann-Whitney U tests by SPSS 16 software at a significance level of P<0.05.

## Results

**Figure-2:**
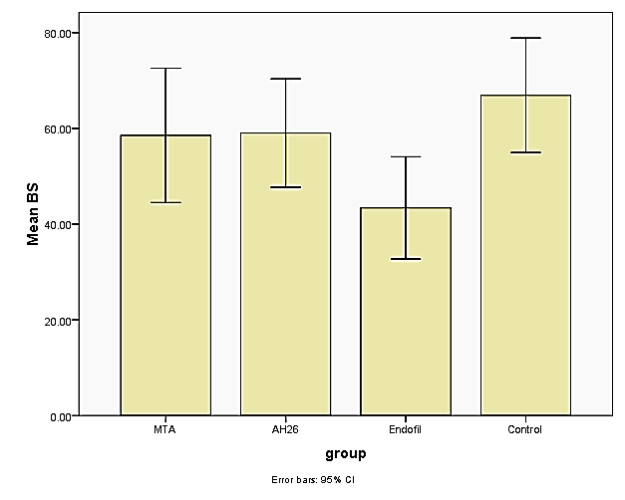
Figure 2. The mean bond strength of study groups

**Table:** Table1. Descriptive statistical results (mean ± standard deviation, SD) for shear bond strength in the studied groups

**Group**	**N**	**Mean** **±** **SD**	**Min.**	**Max.**
MTA	15	58.54 ± 23.21	21	95
AH26	15	59.01 ± 20.42	27	98
Endofill	15	43.38 ± 16.81	16	75
Control	15	66.89 ± 19.79	16	89
Total	60	57.29 ± 21.35	16	98

The results of the nonparametric Kruskal-Wallis test (Table-1) showed a statistically significant difference in mean bond strength in the studied groups (P=0.03), with the highest and the lowest values observed in the control and the Endofill groups, respectively (Figure-2).

The results of the nonparametric Man-Whitney U test showed no significant differences between the mean bond strengths of the two groups (Endofill/AH26 and Endofill/control) (P=0.02 and P=0.1 respectively), but the other groups were not significantly different (P>0.05).

## Discussion

An ideal root canal sealer must adhere firmly to the dentin and filling material; hence, adhesion to the root dentin is an essential feature of root canal sealers [15]. The bond strength of endodontic sealers to dentine is essential for maintaining the seal integrity of root canals [16]. Generally, sealers are divided into eugenol zinc oxide, calcium hydroxide, epoxy resin, glass ionomer, silicon, bioceramic, and MTA-based sealers. These sealers are used in combination with filling materials such as gutta-percha [11].

Root canal sealers are one of the important factors influencing the lifespan of the final restoration [6] and a proper bond of endodontic sealers to the dentin reduces the detachment risk of fillers from the dentin during the restoration and chewing process [17].

In the present study, the shear bond strength of resin composite to coronal dentin contaminated with three sealers (AH26, Endofill, and MTA Fill apex) was investigated. The contamination of the dentin surface with all types of sealers had a significant negative effect on bond strength. In this study, the bond strength was uppermost in the AH26 (resin sealer) group, followed by MTA and Endofill (eugenol-containing sealer), respectively.

Despite the widespread use of eugenol-based sealers (2- methoxy- 4- allyphenol) to fill root canals, these sealers significantly reduce the adhesion to dentine and alter the resin surface polymerization [17]. Mosharraf et al. investigated the effect of endo sealers on the bond strength of fiber-post to the root dentin wall and found that the bond strength in the Endofill group containing the eugenol sealer was lower among all other groups. Eugenol reduces the bond strength by penetrating the dentine tubules due to phenolic components and disruption of polymer chain formation [12]. However, Hagge, et al. concluded that the chemical formulation of endodontic sealers did not affect significantly the retention of posts cemented with resin cement [18].

MTA-based sealers have been introduced to achieve biological properties and suitable seals [19]. According to the manufacturers, the composition of this sealer after mixing includes bismuth, silica, natural resin salicylate resin, and MTA. According to the MTA chemical composition, similarities are expected in the bond strength to the dentine between MTA-based and resin sealers [20]. The high strength of MTA-based sealers relative to eugenol should be related to these similarities.

Forough Reyhani et al. examined the bond strength of three sealers (i.e., MTA, Epiphany, and Dorifill0) to the dentin and reported that resin-based sealers (epiphany) had the highest bond strength, followed by MTA-based and ZOE sealers, respectively [14]. Assman et al. examined the bond strength of dentin in two MTA-based and resin sealers and reported that the highest bond strength belonged to the Endo-CPM sealer and there were no statistically significant differences between MTA Fill apex and AH Plus groups [21]. The weaker results achieved in the MTA-Fillapex group might be due to the weak adhesion of these tag-like structures, which are assumed to compromise the root canal seal. In addition, the resin components in this sealer might negatively affect its bond strength and sealing ability. Also, the resin components in this sealer may negatively affect the bonding strength and its sealing properties [21]. Gurgel-Filho et al. evaluated the pushout bond strength of root canal sealers using Endofill, AHplus, and MTA Fill apex. They observed that the highest and the lowest bond strength belonged to resin and MTA groups, respectively, and there were no statistically significant differences between MTA and Endofill groups [22].

Unlike previous studies, the high bond strength of the MTA Fill apex group was observed in the present study, which can be attributed to the fact that this study was performed on the coronal dentin. In this study, the high strength of resin-based sealers compared to eugenol-based sealers is because the former establishes a covalent bond with the amino group of dentin collagens [23]. Moreover, various studies have attributed the high specificity of resin-based cement results to a low shrinkage during the set process, long dimensional stability, good flow, deep penetration into tubules, and surface irregularities [24].

HM Abada et al investigated the effect of different methods of filling root canals to the root dentin using AH Plus, EndoREZ, and Real Seal sealers and reported that the resin sealer had the highest bond strength in all conditions [25].

In vitro shear tests for measuring bond strength may not be exactly representative of the clinical conditions. Therefore, clinical studies should be performed to validate the results of the present study. Future studies are recommended to use more types of seals from different brands and evaluate the sealer-dentin bond over a longer duration.

## Conclusion

According to the above results, it is concluded that resin sealers have more favorable properties and fewer negative impacts on the composite-to-dentin bond. Therefore, they seem to be a suitable material for use in root canal treatments.

## Conflict of Interest

None.
